# Soft Coral *Sarcophyton* (Cnidaria: Anthozoa: Octocorallia) Species Diversity and Chemotypes

**DOI:** 10.1371/journal.pone.0030410

**Published:** 2012-01-17

**Authors:** Satoe Aratake, Tomohiko Tomura, Seikoh Saitoh, Ryouma Yokokura, Yuichi Kawanishi, Ryuichi Shinjo, James Davis Reimer, Junichi Tanaka, Hideaki Maekawa

**Affiliations:** 1 Graduate School of Science and Engineering, University of the Ryukyus, Nishihara, Okinawa, Japan; 2 Center of Molecular Biosciences, Tropical Biosphere Research Center, University of the Ryukyus, Nishihara, Okinawa, Japan; 3 Department of Chemistry, Biology, and Marine Science, University of the Ryukyus, Nishihara, Okinawa, Japan; 4 Department of Physics and Earth Sciences, University of the Ryukyus, Nishihara, Okinawa, Japan; 5 Rising Star Program, TRO-SIS, University of the Ryukyus, Nishihara, Okinawa, Japan; Biodiversity Insitute of Ontario - University of Guelph, Canada

## Abstract

Research on the soft coral genus *Sarcophyton* extends over a wide range of fields, including marine natural products and the isolation of a number of cembranoid diterpenes. However, it is still unknown how soft corals produce this diverse array of metabolites, and the relationship between soft coral diversity and cembranoid diterpene production is not clear. In order to understand this relationship, we examined *Sarcophyton* specimens from Okinawa, Japan, by utilizing three methods: morphological examination of sclerites, chemotype identification, and phylogenetic examination of both *Sarcophyton* (utilizing mitochondrial protein-coding genes MutS homolog: *msh1*) and their endosymbiotic *Symbiodinium* spp. (utilizing nuclear internal transcribed spacer of ribosomal DNA: ITS- rDNA). Chemotypes, molecular phylogenetic clades, and sclerites of *Sarcophyton trocheliophorum* specimens formed a clear and distinct group, but the relationships between chemotypes, molecular phylogenetic clade types and sclerites of the most common species, *Sarcophyton glaucum*, was not clear. *S. glaucum* was divided into four clades. A characteristic chemotype was observed within one phylogenetic clade of *S. glaucum*. Identities of symbiotic algae *Symbiodinium* spp. had no apparent relation to chemotypes of *Sarcophyton* spp. This study demonstrates that the complex results observed for *S. glaucum* are due to the incomplete and complex taxonomy of this species group. Our novel method of identification should help contribute to classification and taxonomic reassessment of this diverse soft coral genus.

## Introduction

Soft corals (Cnidaria: Anthozoa: Octocorallia) often equal or exceed the total coverage of scleractinian corals in coral reef ecosystems [1]–[4], and as dominant space-occupiers, important structural components of coral reef communities, and contributors to coral reef biomass [4], [5], have been the subjects of biological studies since the nineteenth century.

The subclass Octocorallia includes soft corals, gorgonians, and sea pens. Most soft corals belong to the order Alcyonacea, which is comprised of the families Xeniidae, Nephtheidae, and Alcyoniidae. The family Alcyoniidae contains the genera *Sinularia*, *Lobophytum* and *Sarcophyton*, and members of this group are among the dominant benthic organisms in the coral reefs in Okinawa and other Pacific Ocean areas [1], [2], [4], [6]. *Sarcophyton* species are very hardy and are dominant in many coral reef areas. *Sarcophyton* species are characterized by a distinct sterile stalk, a broad, flared, smooth, mushroom-shaped top called a capitulum, and by the shape of their sclerites, which are found in the interior coenenchymal tissue of the colony.

Most soft coral classification and identification has traditionally been carried out by sclerite characterization. Verseveldt [7] revised the classification of *Sarcophyton* after gross morphological and microscopic examination of *Sarcophyton* species' type specimens. Since the taxonomic revision by Verseveldt [7], who considered *Sarcophyton* to contain 35 valid species, an additional six species of *Sarcophyton* have been described [8]–[13].

Recently, McFadden et al. [14] reported on the utility of mitochondrial protein-coding gene MutS homolog (*msh1*) sequences for *Sarcophyton* and *Lobophytum* species identification. The study showed that within *Sarcophyton*, specimens initially identified as *Sarcophyton glaucum* by morphology could be divided into six very distinct genetic clades, suggesting that this morphologically heterogeneous species is actually a complex of cryptic species [14].

The soft coral genera *Lobophytum* and *Sarcophyton* are known to have many secondary metabolites [15]–[17]. Secondary metabolites in soft corals of *Sarcophyton* have been well characterized with the advancement of instrumental analyses over the past four decades. The soft coral egg-specific secondary metabolite PGA_2_ and some diterpenes have been shown to cause contractions of soft coral polyps and the expulsion of eggs during spawning [18], and similar phenomenon by a secondary metabolite (sarcophytoxide) has been reported from *Sarcophyton glaucum* [19]. These examples indicate one reproductive isolation factor may be due to chemical signals, and that secondary metabolites may have important function. In addition, some metabolites are toxic and used in competition for space with scleractinian corals [20], and it is believed that octocorals release chemical substances into the water as a commonly used strategy to inhibit growth and survival of their neighbors [21]. Furthermore, it is known in *Sarcophyton glaucum* that secondary metabolites such as sarcophytoxide cause allelopathic effects [19]. Thus, by focusing attention on secondary metabolites it may be possible to better understand the environmental role of soft corals in tropical waters.

One molecule, sarcophytol A, has attracted attention due to its antitumor promoting activity [22]. As sarcophytol A was discovered from *Sarcophyton* collected at Ishigaki Island, Okinawa, southern Japan, researchers have investigated the chemical activity and three-dimensional structure of the chemical [23]–[25]. Additionally, Koh et al. [26] investigated the distribution of *Sarcophyton* species containing sarcophytol A in Okinawa, and their study indicated that composition of cembranoids in *Sarcophyton* is not related with morphologically identified species. Subsequently, it was found that two species, *Sarcophyton trocheliophorum* and *Sarcophyton crassocaule*, appeared to be the source organisms of sarcophytol A [27], and not only *Sarcophyton glaucum* as originally reported. During this study, it was also noted that *Sarcophyton glaucum*'s chemical content varied to a large degree and it was concluded there are at least nine chemotypes within *S. glaucum* [27].

Thus, it is difficult to conclusively identify the source *Sarcophyton* species of secondary metabolites from past studies' data. Furthermore, secondary metabolites obtained from marine organisms are often derived from symbiotic algae and/or symbiotic bacteria [28], [29]. *Sarcophyton* spp. contain endosymbiotic dinoflagellate zooxanthellae (*Symbiodinium* spp.), but no study has yet examined whether there are any relations between soft coral chemotype, genotype (molecular phylogenetic clade), and their *Symbiodinium*, despite many studies demonstrating the diversity of *Symbiodinium* spp. found within different coral reef invertebrate hosts [30], [31].

In order to more fully understand the relationship between secondary metabolites and *Sarcophyton* species, in this study we examined specimens from Okinawa, Japan utilizing three methods; 1) novel morphological examination of sclerites, 2) chemotype identification, and 3) phylogenetic examination of both *Sarcophyton* (utilizing *msh1* sequences) and their endosymbiotic *Symbiodinium* spp. (ITS-rDNA sequences). From our results, we examine the production pattern of secondary metabolites by *Sarcophyton* species, and theorize on the mechanism behind such varied secondary metabolite production in this soft coral genus.

## Results

### Molecular phylogeny using mitochondrial *msh1* sequences

**Figure 1:**
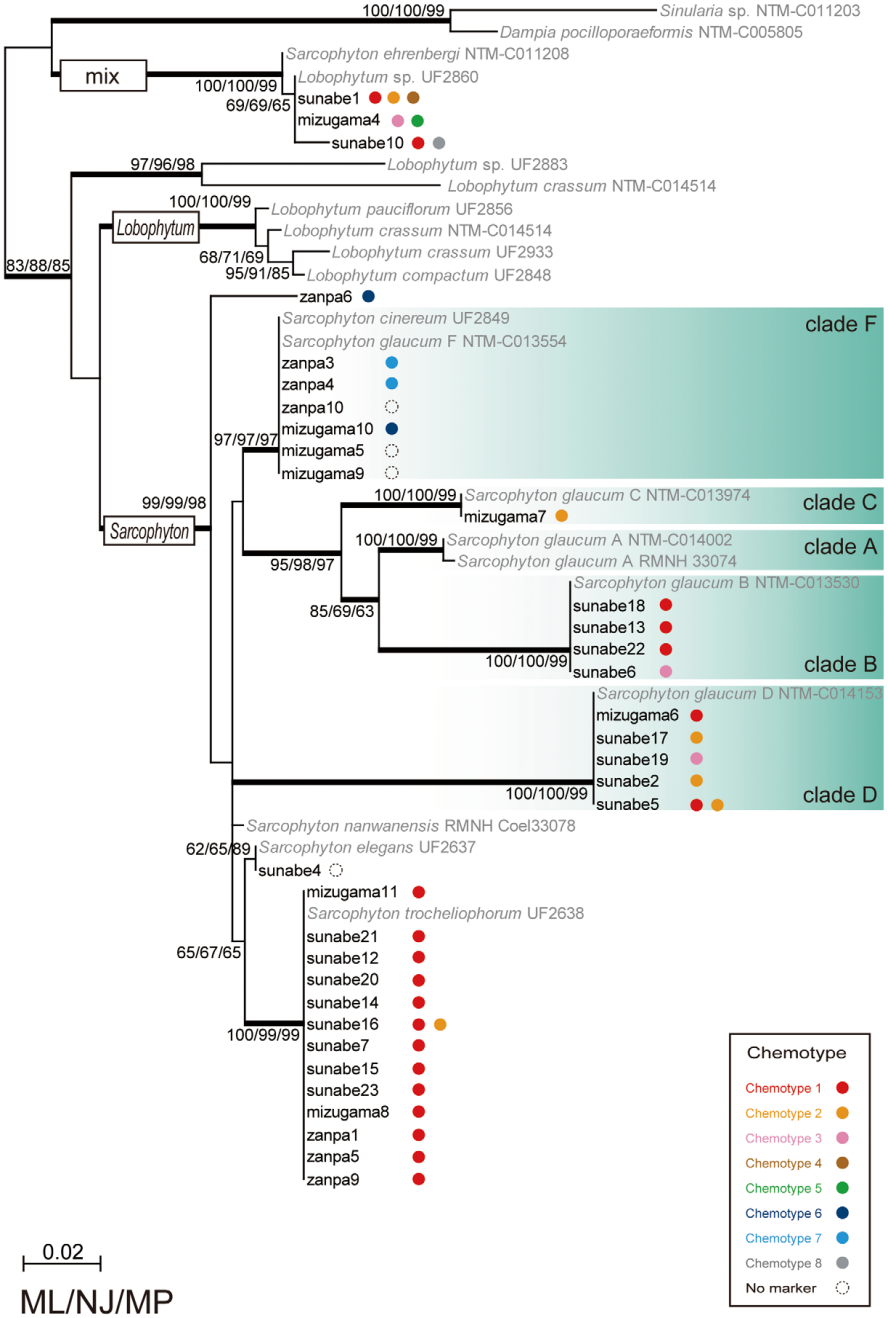
Phylogenetic analyses of *Sarcophyton* species and relationship with chemotypes. Phylogentetic tree of an alignment of utilizing mitochondrial protein-coding genes MutS homolog *msh1* sequences for *Sarcophyton* specimens constructed by the maximum likelihood (ML) method. Values at branches represent ML, neighbor-joining (NJ) and maximum parsimony (MP) method bootstrap values, respectively. Monophylies with more than 95% Bayesian posterior probabilities are shown by thick branches. Sequences in bold without GenBank accession numbers are *msh1* sequences newly obtained in this study. Color dots indicate different chemotypes as described in this study. For chemotype information see Figure 2 and for specimen information see Table 1.

Most specimens' sequences (n = 31) were found to clearly belong to a genus *Sarcophyton* clade, while three specimens (Sunabe 1, 10, and Mizugama 4) were classified into a mixed clade consisting of previously reported sequences from both *Sarcophyton* and *Lobophytum* specimens (Fig. 1). All sequences could be aligned unambiguously. Of the 31 “clear” *Sarcophyton* sequences, 13 sequences were identified as being from *Sarcophyton trocheliophorum*, 16 from *Sarcophyton glaucum*, one from *Sarcophyton elegans*, with the remaining one sequence not assignable to any previously reported species group. All putative *Sarcophyton trocheliophorum* specimens had exactly the same sequence regardless of sampling location. *Sarcophyton glaucum* has previously been divided into six phylogenetic clades A–F [14], and specimens from this study belonged to four of these clades: four sequences within clade B *sensu* McFadden et al. [14], one within C, five within D, and six within F.

### Major compound analyses: Cembrene diterpenes

**Figure 2:**
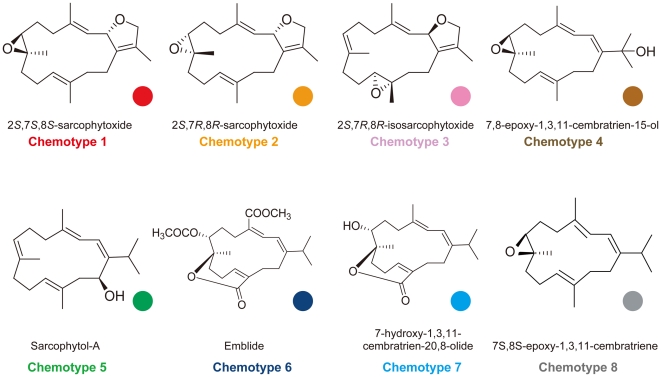
Structures of cembranes from *Sarcophyton* species identified in this study. Colored dots next to each cembrane are the same as in other figures.

In total eight cembranoid diterpenes were identified (chemotypes 1–8) (Fig. 2). The abundance of each chemotype at each collection site is shown in Table 1. Among the detected chemotypes, 20 specimens of chemotype 1 (2*S*,7*S*,8*S*-sarcophytoxide) were most abundant, followed by chemotype 2 (2*S*,7*R*,8*R*-sarcophytoxide) and chemotype 3 (2*S*,7*R*,8*R*-isosarcophytoxide). The cembrenes found from the 34 specimens were as follows: chemotype 1 - 2*S*,7*S*,8*S*-sarcophytoxide, 20 specimens (Sunabe 1, 5, 7, 10, 12, 13, 14, 15, 16, 18, 20, 21, 22, 23, Zanpa 1, 5, 9, Mizugama 6, 8, 11); chemotype 2 - 2*S*,7*R*,8*R*-sarcophytoxide, six specimens (Sunabe 1, 2, 5, 16, 17, Mizugama 7); chemotype 3 - 2*S*,7*R*,8*R*-isosarcophytoxide, three specimens (Sunabe 6, 19, Mizugama 4); chemotype 4–7,8-epoxy-1,3,11-cembratrien-15-ol, one specimen (Sunabe 1); chemotype 5 - Sarcophytol A, one specimen (Mizugama 4); chemotype 6 - Emblide, two specimens (Zampa 6, 10); chemotype 7 - 7-hydroxy-1,3,11-cembratrien-20,8-olide, two specimens (Zampa 3, 4); chemotype 8 - 7*S*,8*S*-epoxy-1,3,11-cembratriene (Sunabe 10).

**Table 1:** Summary of field sites and chemotypes.

		Chemotype No								
Sampling site	Sample size	1	2	3	4	5	6	7	8	9
Sunabe	23	14	5	2					1	1
Zanpa	10	3		1	1		3	2		
Mizugama	11	3	1	2		1				
Total	44	20	6	5	1	1	3	2	1	1

For chemotype information see Figure 2.

Specimens obtained from three field sites (April 2007-November 2007) were collected at depths of 5–20 m (see Materials and Methods).

All specimens of *Sarcophyton trocheliophorum* included the same chemotype, chemotype 1. Specimens of *Sarcophyton glaucum* clade F included two chemotypes, 6 and 7. Chemotypes 6 and 7 have lactone function and could be easily distinguished from the other chemotypes. Specimens of *Sarcophyton glaucum* clade B included only chemotype 1 with the exception of Sunabe 6, which had chemotype 3. Specimens of *Sarcophyton glaucum* clade D included chemotypes 1, 2 and 3. Though clades B and D included different chemotypes, those chemotypes had similar chemical isomerism, containing dihydro furan and epoxy groups. *Sarcophyton glaucum* clades B and D, and *Sarcophyton trocheliophorum* included similar chemotypes despite of clearly belonging to different clades.

The phylogenetic group classified to the “mixed” *Sarcophyton*-*Lobophytum* clade includes six chemotypes and in this clade no relationship between chemotype and molecular phylogenetic clade was apparent.

### Morphological analyses

*Sarcophyton* spp. were examined morphologically by observing colony growth form and sclerite characters. We examined sclerites with a light microscope for species identification. Sclerite identification followed Verseveldt [7], with “clubs” being club sclerites in the surface layer of the disc.

**Figure 3:**
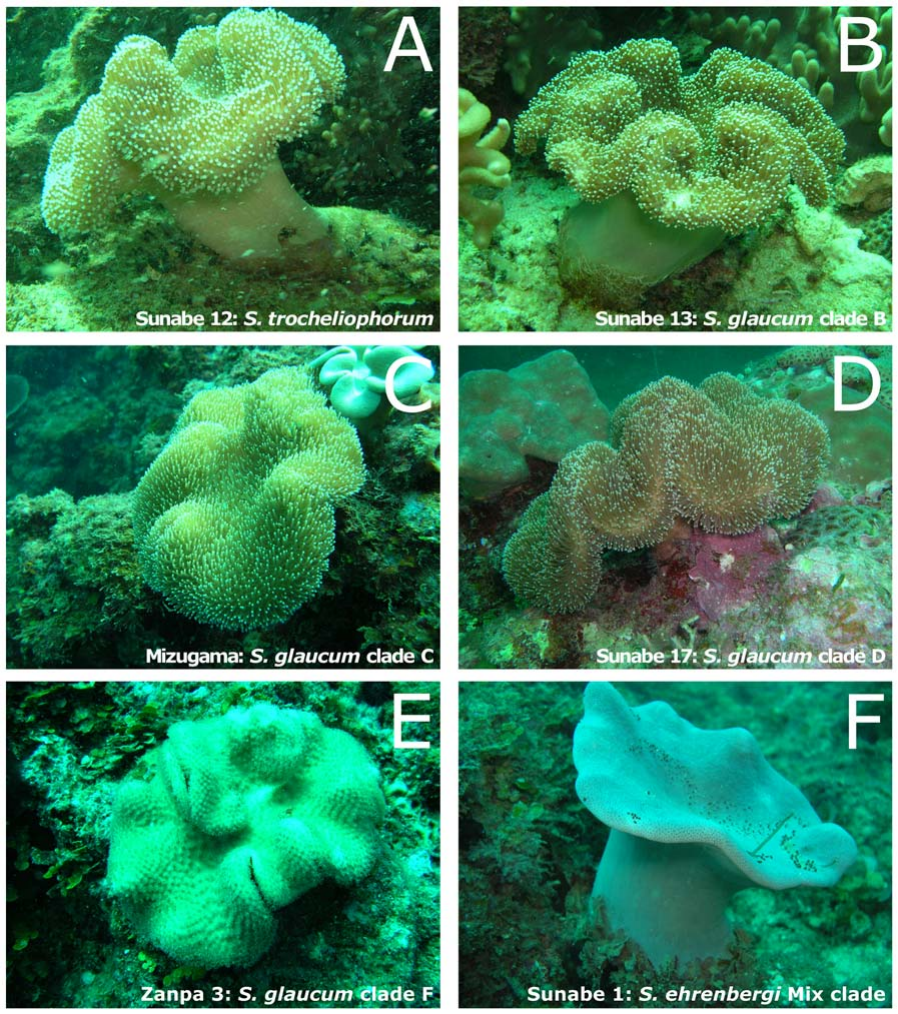
*In situ* photographs of colonies of *Sarcophyton*. **A**. *Sarcophyton trocheliophorm*, Sunabe 12. **B**. *Sarcophyton glaucum* clade B, Sunabe 13. **C**. *Sarcophyton glaucum* clade C, Mizugama 7. **D**. *Sarcophyton glaucum* clade D, Sunabe 17. **E**. *Sarcophyton glaucum* clade F, Zanpa 3. **F**. *Sarcophyton ehrenbergi* mixed clade, Sunabe 1.

*Sarcophyton* colonies have a mushroom-shaped polypary consisting of a smooth and marginally folded disc, which projects beyond a clearly differentiated base or stalk (Fig. 3). Surface sclerites were usually long-handled clubs with poorly differentiated heads and fairly sparse, simple ornamentation. *Sarcophyton glaucum* and closely related *Sarcophyton cinereum* were identified by the presence of moderately ornamented clubs [7] though there was a range of development of the warts and in the sclerites' length. *Sarcophyton glaucum* possessed clubs usually 0.10–0.17 mm in length, and rarely more than 0.35 mm in length, with the clubs having low, rounded processes. *Sarcophyton cinereum* possessed clubs usually 0.15–0.2 mm long, with the longest measuring 0.70 mm, and the clubs had warty heads. However, using existing identification keys [7], sclerite differences between *Sarcophyton glaucum* and *Sarcophyton cinereum* could not be determined.

**Figure 4:**
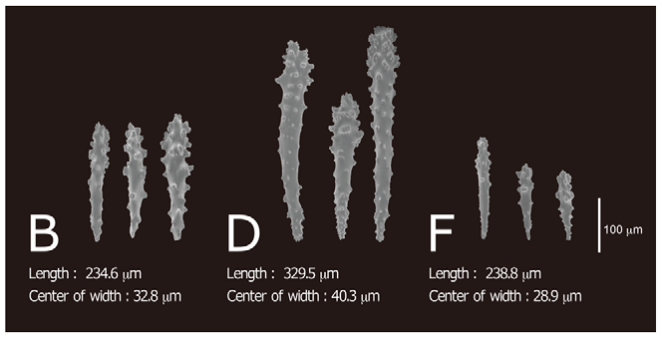
Sclerites of *Sarcophyton glaucum* clades B, D and F, and their averages length and width. All sclerites shown are surface sclerites; Clade B obtained from specimens Sunabe 6 and Sunabe 13; Clade D from Sunabe 2, Sunabe 19; Clade F from Zanpa 3, Mizugama 5, Mizugama 9. Images were taken using a scanning electron microscope.

After obtaining a phylogenetic tree based on *msh1* sequences, we re-examined the sclerites with a scanning electron microscope (SEM; S-3500N: Hitachi High-Technologies). Sclerites from the capitulum surface of *Sarcophyton* were usually long-handled clubs with poorly differentiated heads and fairly sparse and simple ornamentation. This was seen particularly in specimens of the most dominant species, *Sarcophyton glaucum*. According to the molecular phylogeny, *Sarcophyton glaucum* was comprised of four clades and we therefore compared the sclerites of clades B, D, F of *S. glaucum* (Fig. 4).

Clade D sclerites were longer than sclerites of clades B and F (nested ANOVA, length, D>F = B, P<0.05), and the warts were comparatively concentrated on the head. The sclerites of clade F were comparatively short and slim (nested ANOVA, width, D>B>F, P<0.05). These values are summarized in Table 2.

**Table 2:** Mean and standard deviation (SD) of length and width (mm) from sclerites of each phylogenetic clade.

Clade	*n*	Length	Width
		mean±SD*	mean±SD*
Clade B	4	0.2289±0.0602^a^	0.0282±0.0042^a^
Clade D	5	0.3146±0.0820^b^	0.0348±0.0068^b^
Clade F	5	0.2096±0.0739^a^	0.0238±0.0051^c^

*Values were calculated based on pooled data.

Letters following SD values indicate different statistical significances in nested ANOVA.

Each specimen had 100 sclerites examined.

**Figure 5:**
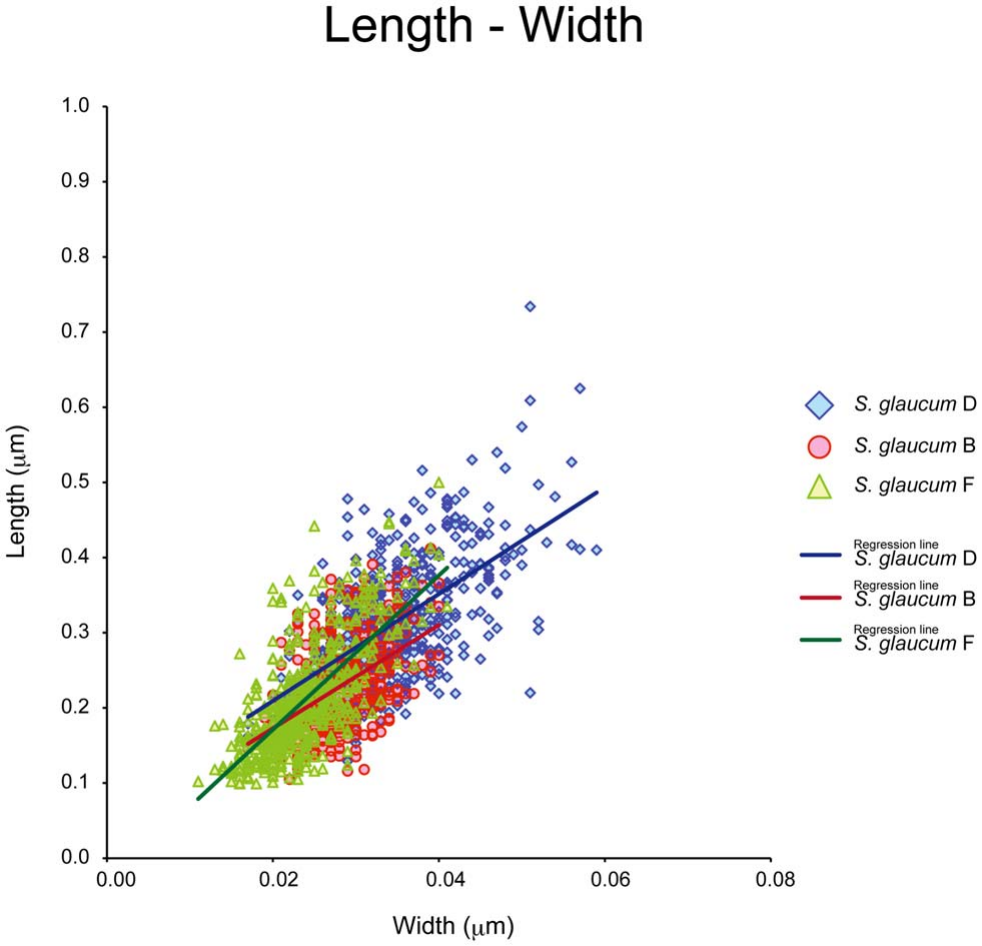
Scatter plot and regression line of length and width of sclerites of each *Sarcophyton glaucum* clade. Horizontal axis: width of sclerites, vertical axis: length of sclerites.

Analyses of covariance (ANCOVA) showed that the slope of regression line between length and width was statistically different between clade B and clade F (*P*<0.001), and between clade D and clade F (*P*<0.001), but not between clade B and clade D (*P* = 0.78). However, the adjusted mean significantly differed between clade B and D (*P*<0.001) (Fig. 5).

*Sarcophyton trocheliophorum* could be easily identified by the presence of torch-shaped small sclerites in the surface of the capitulum (Fig. S1)

### Phylogenetic analysis of *Symbiodinium* ITS-rDNA

Most obtained *Symbiodinium* ITS-rDNA sequences were found to match most closely with *Symbiodinium* clade C *sensu* LaJeunesse [30] with 96–100% identity (data from NCBI GenBank) and all novel sequences from this study belonged to clade C, consisting of numerous sequences closely related to type C1 *sensu* LaJeunesse [30]. Chemotypes were graphed onto to the resulting *Symbiodinium* ITS-rDNA phylogenetic tree (Fig. S2), but no relation between *Symbiodinium* ITS-rDNA and chemotype was discernable.

## Discussion

The molecular phylogenetic tree based on *msh1* revealed two large and very well-supported clades; one including only *Sarcophyton* and the other a mix of *Sarcophyton* and *Lobophtum*). Similar to a previous report on intergeneric diversity in *Sarcophyton* [14] clades of *Sarcophyton glaucum* were observed. Uniquely, correlations between *Sarcophyton* chemotypes and molecular phylogenetic clades were observed in this study.

*Sarcophyton glaucum* specimens formed at least four distinct subclades (B, C, D, and F). Clade F consisted of chemotypes 6 and 7, which contain emblide and an analogue encompassing a ε-lactone ring in their structure and therefore clade F likely retains a different set of biosynthetic pathways from the other *Sarcophyton glaucum* clades. Clade B consisted of chemotypes 1 and 3, clade C of chemotype 2, and clade D of chemotypes 1, 2, and 3. By examining the structures of these chemotypes by high performance liquid chromatography (HPLC) and Nuclear Magnetic Resonance (NMR), it was determined that the structures of chemotypes 1, 2, and 3 are isomeric. The structures of chemotype 1 and chemotype 2 were diastereomeric, while those of chemotype 3 and chemotypes 1 and 2 were structurally isomeric (or regioisomeric). These results mean that these clades likely share similar biosynthetic or oxidative enzymes involved in the production of cembranoids. Additionally, all three examined specimens (Sunabe 1, 10, Mizugama 4) belonging to the “mixed clade” of *Sarcophyton* and *Lobophytum* were also found to have mixed chemotypes (Sunabe 1 - chemotypes 1, 2 and 4; Sunabe 10 - chemotypes 1 and 8; Mizugama 4 - chemotypes 3 and 5). This situation could potentially be caused by interspecific hybdrization, as previously suggested by McFadden et al. [14].

The current confused situation of *Sarcophyton* taxonomy is caused by the combination of three problems; 1) relatively few diagnostic morphological characters available for study in *Sarcophyton*, 2) our present lack of understanding of intraspecific variation of diagnostic morphological characters within this genus, and 3) a historical lack of taxonomic and ecological work on *Sarcophyton* [14]. Therefore, molecular phylogenetic analyses alone are not yet sufficient to clearly identify *Sarcophyton* specimens. However, our results suggest that detailed, morphometric examinations of sclerites may greatly aid in clarifying the meaning of molecular phylogenetic analyses of *Sarcophyton* species. The outcome of chemotype and statistical analyses of sclerites fully supported the molecular phylogenetic analyses' results. In this study, sclerite examination detected differences between three *Sarcophyton glaucum* subclades. Therefore, we expect that further in-depth examinations may yield additional diagnostic morphological characters. Based on the all results of this study, we propose that clades B, D and F of *Sarcophyton glaucum* should be formally classified into independent species in the future.

All *Sarcophyton* specimens contained *Symbiodinium* clade C *sensu* LaJeunesse [30], belonging to closely related ITS-rDNA types. However, in total eight chemotypes were found within *Sarcophyton* specimens, and there was no meaningful correlation between *Symbiodinium* and chemotype. Further support can be found from the azooxanthellate soft coral genus *Dendronephthya* soft coral, in which several types of diterpenes are found [32] despite the lack of *Symbiodinium*. Thus, we believe it is unlikely *Symbiodinium* is involved in the synthesis of the chemical examined in this study.

It is noteworthy that some interspecific, different clades have similar secondary metabolites. We suspect that the secondary metabolites of *Sarcophyton* may have some kind of relationship with their environment although this was not examined in this study. By focusing on the relationship between chemotype and sampling site, some indicative patterns are apparent. Chemotypes 6 and 7 were only found at Zanpa regardless of *Sarcophyton* clade. Clades B, C, D of *Sarcophyton glaucum* were not collected in Zanpa, and clade F was not obtained in Sunabe. At Mizugama, located between Sunabe and Zanpa on the west coast of Okinawa, all clades except clade B were present. Clades B, C and D have chemotypes 1, 2 and 3, and were dominant at Sunabe, while clade F with chemotypes 6 and 7 was dominant at Zanpa. From these results, it appears that chemotypes of *Sarcophyton glaucum* are related to both environment (sampling location) and species-group/molecular phylogenetic clade. However, *Sarcophyton trocheliophorum* had similar chemotypes regardless of sampling site, and it is not known if our theory is therefore applicable to *Sarcophyton* species in general.

It is already known that soft corals have significant diversity of secondary metabolites and it has been speculated that such chemicals are used for allelopathic effects in soft coral [19], [33]–[35]. However, the function of secondary metabolites could be related to survival in different environments, as our results demonstrate a relationship between species, sampling site, and secondary metabolite variation. In *Sinularia*, it has been reported that compounds may be influenced by the environment [36]. This theory should be investigated in the near future.

Currently, research on soft coral ecological, reproductive, and behavioral differences has not progressed well as soft coral taxonomy is confused, and the confused taxonomy in turn hinders studies on these topics. We suggest that research on secondary metabolite variation could be an important key in understanding soft coral ecology, reproduction, behavioral differences, and classification. We consider it possible that variation in secondary metabolites may be related to environmental adaptation and adaptive evolution in soft corals.

## Materials and Methods

### Collection of specimens

**Figure 6:**
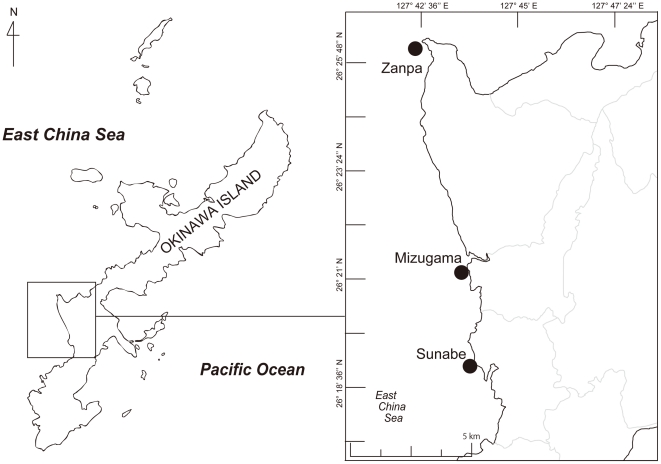
Map of collection sites of specimens examined in this study. Okinawa Island is located in southern Japan.

*Sarcophyton* specimens were collected from a depth range of 5–20 m by SCUBA at three locations (Sunabe 26°19′N; 127°44′E, Zanpa 26°26′N; 127°42′E, and Mizugama 26°21′N; 127°44′E) on the west coast of Okinawa Island in 2007 (Fig. 6). No specific permits were required for the described field studies. The three locations examined in this study are popular public diving spots and are not privately owned, and are not in a protected area. This study did not involve any endangered or protected species. The numbers of specimens from each collection site were: 23 from Sunabe, 10 from Zampa, and 11 from Mizugama, respectively. Specimens were designated Sunabe 1 to Sunabe 23, Zampa 1 to Zampa 10, Mizugama 1 to Mizugama 11. Specimens were separated into subsamples for chemical analyses, morphological analyses, and genetic analyses. Genetic subsamples were fixed in 70–99% cold ethanol and kept at −30°C until DNA extraction.

### DNA extraction

Each genetic subsample was cut into small pieces of approximately 20 mg, and treated with 20 mL proteinase K in 180 mL ALT buffer for 4–6 h at 56°C. Then, total genomic DNA was extracted from each specimen using a spin-column DNeasy Animal DNA Extraction kit following the manufacturer's protocol (QIAGEN, Tokyo, Japan).

### PCR analyses of mitochondrial *msh1*: *Sarcophyton*

The 5′ end of the mitochondrial *msh1* gene was amplified by PCR using the primers ND42599F (5′-GCCATTATGGTTAACTATTAC-3′) and Mut-3458R (5′-TSGAGCAAAAGCCACTCC-3′) [14]. The PCR reaction used 20 pmol of each primer, 4 mL of dNTP mix, 0.25 mL of *Taq* polymerase, 5 mL of *Taq* Buffer, and 1mL of raw genomic DNA. Several samples were cloned into the pCR2.1 vector of the TOPO TA Cloning Kit (Invitrogen, Carlsbad, CA, USA). All primers were anchored in an adjacent mitochondrial gene to prevent amplification of genes from nuclear or symbiont (*Symbiodinium* spp.) genomes. PCR products were sequenced using an ABI PRISM Big Dye Terminator cycle sequencing kit Ver. 3.1 (Applied Biosystems, Foster City, CA) with a DNA sequences system (Model 3100 or 3130, Applied Biosystems).

### PCR analyses of ITS-rDNA: *Symbiodinium*

The internal transcribed spacer of ribosomal DNA (ITS-rDNA) was amplified using primers ITS-4 (5′-TCCTCCGCTTATTGATATGC-3′) [37] and zooxanthellae-specific zITSf (5′-CCGGTGAATTATTCGGACTGACGCAGT-3′) [38], [39]. The purified PCR-amplified DNA fragments were cloned into the pCR2.1 vector of the TOPO TA Cloning Kit (Invitrogen, Carlsbad, CA, USA). Several clones of ITS-1 - 5.8S rDNA - ITS-2 from each site were sequenced using an ABI PRISM Big Dye Terminator cycle sequencing kit Ver. 3.1 (Applied Biosystems, Foster City, CA) with a DNA sequencing system (Model 3100 or 3130, Applied Biosystems).

Novel sequences from this study are available at GenBank under the accession numbers AB665446-AB665479 (*msh1*) and AB665603-AB665723 (ITS-rDNA) (Table S3).

### Phylogenetic analyses

Nucleotide sequences were assembled and proofread using Sequence Scanner v1.0 software, and aligned using MEGA 4 [40]. Members of the genera *Sinularia* and *Dampia* were included as outgroup taxa of the *msh1* alignment (Table S1). For the *Symbiodinium* ITS-rDNA alignment, *Symbiodinium* sp. 1591 type C91 (GenBank accession number AJ291519) [41] was included as the outgroup (Table S2). Consequently, two alignments were generated, one of soft coral *msh1* sequences (34 taxa; 735 base pairs) and one of *Symbiodinium* ITS-rDNA sequences (121 taxa; 704 base pairs). Both alignments are available upon request from the corresponding author. The datasets of *msh1* alignments and ITS-rDNA alignments were separately subjected to maximum-likelihood (ML) and neighbor-joining (NJ) [42] analyses. In addition, phylogenetic trees of *msh1* were obtained using MrBayes and maximum parsimony method (MP) analyses. ML analyses were performed using PhyML online web server [43]. PhyML was performed using an input tree generated by BIONJ with the general time-reversible model [44] of nucleotide substitution incorporating invariable sites and a discrete gamma distribution (eight categories) (GTR+I+Γ). The proportion of invariable sites, a discrete gamma distribution and base frequencies of the model were estimated from the dataset. PhyML bootstrap trees (1000 replicates) were constructed using the same parameters as the individual ML trees. The NJ tree was constructed using maximum composite likelihood model. Support for NJ branches was tested by bootstrap analysis of 1000 replicates. The NJ and MP methods were conducted using MEGA 4. Bayesian phylogenetic analyses were conducted using MrBayes 3.1.2 [45] with a GTR+I+Γ model run for 10,000,000 generations with sampling of trees at 100-generation intervals (burn-in = 1500 generations).

### Major compound identification

Specimens for chemical analyses were extracted with acetone two times, and the acetone solution was then filtered and concentrated under vacuum, with the residual material was partitioned between CH_2_Cl_2_ and water. The lipophilic portion was subjected for chemical analyses. Each CH_2_Cl_2_ extract was analyzed first with thin layer chromatography (TLC) and ^1^H NMR (Nuclear Magnetic Resonance) to examine whether a dominant marker cembrane existed or not. Then, the presence of major cembranoid was confirmed qualitatively by high performance liquid chromatography (HPLC) equipped with a photodiode array detector using an ODS column with linear gradient elution profile. ^1^H and ^13^C NMR spectra ware taken on a Jeol A-500 by dissolving extracts or pure compound in CDCl_3_ using tetramethylsilane as an internal standard.

### Structure identification of cembrane diterpenes

Observed compounds (designated compounds 1–8) were identified by comparing NMR spectral data with those previously published after obtaining nearly pure material with separation on column, TLC, or HPLC.

### Analysis of sclerites

From the capitulum of each specimen, a small portion (0.4 cm^2^) was removed and treated with 10% sodium hypochlorite. After removal of excess hypochlorite with water, sclerites were observed under light microscope at ×400 magnification. Subsequently, spicules for *Sarcophyton glaucum* and *Sarcophyton trocheliophorum* were observed with a scanning electron microscope (SEM; S-3500N: Hitachi High-Technologies) to examine sclerite size and potential morphological differences between different specimens. For each specimen, morphological traits (length and center width) of sclerites (n = 100) were measured with ImageJ 1.44 software (NIH).

A nested ANOVA was used to examine the effect of genetic clade on morphological traits (length or width of sclerites). Firstly, a nested ANOVA was conducted using data from all clades. Secondary, if the effect of clade was significant (*P*<0.05), nested ANOVA was performed for each of all possible pairs of clades (i.e. clade F vs B, B vs D, or D vs F). P-values from the analyses were adjusted with Bonferroni correction. Analyses of covariance (ANCOVA) were performed to examine the difference in ratio of length and width of sclerites among clades (*Sarcophyton glaucum* B, D, F). We evaluated discrepancies in *P* values for each data set, considering significant differences at *P* values of 0.001. Statistical analysis was performed using R software (version 2.12.0; R Foundation for Statistical Computing, Vienna, Austria).

## Supporting Information

Figure S1 **Sclerites of** ***Sarcophyton trocheliophorum*** **.** Surface sclerites of Sunabe 7 are shown. Images were taken using a scanning electron microscope.
(TIF)

Figure S2 **Phylogenetic analyses of** ***Symbiodinium*** **spp.** Neighbor-joining (NJ) tree of an alignment of nuclear internal transcribed spacer of ribosomal DNA (ITS rDNA) sequences of symbiotic *Symbiodinium* dinoflagellates (clade C) associated with genus *Sarcophyton*. Values at branches represent NJ and maximum likelihood (ML) bootstrap values, respectively. (−) indicates bootstrap values <50%. Sequences in bold without GenBank accession numbers are ITS-rDNA sequences obtained in this study. Colored dots indicate chemotypes as in Figure 2.
(TIF)

Table S1 List of mitochondrial protein-coding gene MutS homolog *msh1* sequences from previous studies used in phylogenetic analyses in the present study. Species, GenBank accession numbers, geographic origin, latitude and longitude, and collection date are also shown.
(DOC)

Table S2 List of internal transcribed spacer of ribosomal DNA (ITS-rDNA) sequences from previous studies used in phylogenetic analyses in the present study. Species, GenBank accession numbers, geographic origin, and host species are shown.
(DOC)

Table S3 Collection information for specimens included in molecular phylogenetic clade.
(DOC)
