# Supplementary figures and images for: Soft Coral Sarcophyton (Cnidaria: Anthozoa: Octocorallia) Species Diversity and Chemotypes
Source: PLoS One. 2012 Jan 17;7(1):e30410. doi: 10.1371/journal.pone.0030410 (PMC3260304; doi:10.1371/journal.pone.0030410)

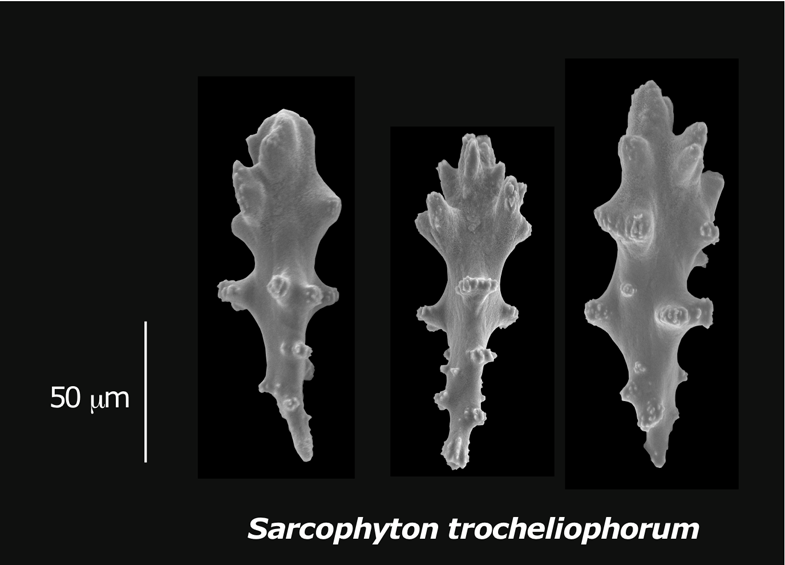

Supplement: Figure S1 — Sclerites of Sarcophyton trocheliophorum . Surface sclerites of Sunabe 7 are shown. Images were taken using a scanning electron microscope. (TIF) [file pone.0030410.s001.tif]

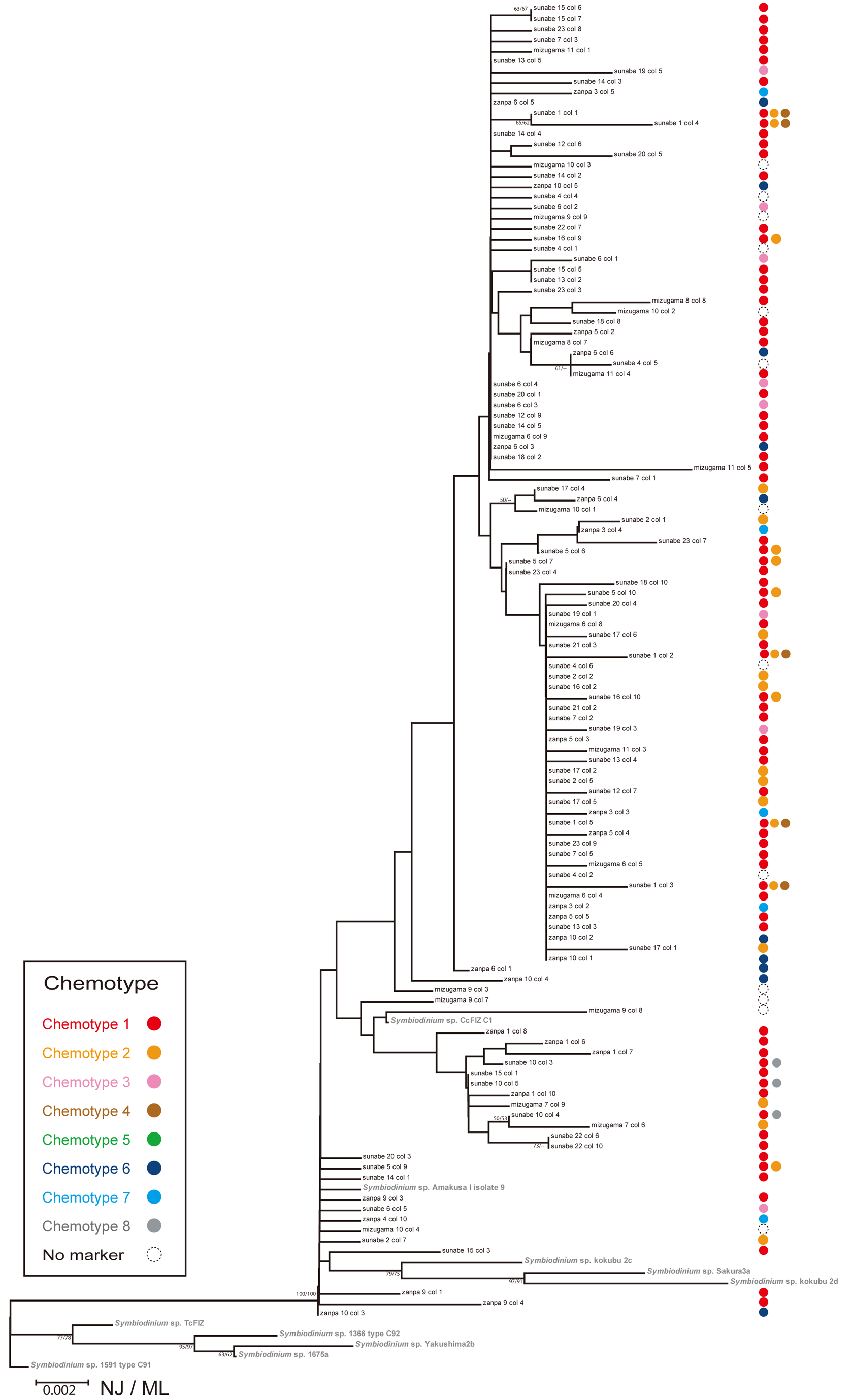

Supplement: Figure S2 — Phylogenetic analyses of Symbiodinium spp. Neighbor-joining (NJ) tree of an alignment of nuclear internal transcribed spacer of ribosomal DNA (ITS rDNA) sequences of symbiotic Symbiodinium dinoflagellates (clade C) associated with genus Sarcophyton. Values at branches represent NJ and maximum likelihood (ML) bootstrap values, respectively. (−) indicates bootstrap values <50%. Sequences in bold without GenBank accession numbers are ITS-rDNA sequences obtained in this study. Colored dots indicate chemotypes as in Figure 2. (TIF) [file pone.0030410.s002.tif]
