# Supplementary material for: Soft Coral Sarcophyton (Cnidaria: Anthozoa: Octocorallia) Species Diversity and Chemotypes
Source: PLoS One. 2012 Jan 17;7(1):e30410. doi: 10.1371/journal.pone.0030410 (PMC3260304; doi:10.1371/journal.pone.0030410)
Supplement: Table S1 — List of mitochondrial protein-coding gene MutS homolog msh1 sequences from previous studies used in phylogenetic analyses in the present study. Species, GenBank accession numbers, geographic origin, latitude and longitude, and collection date are also shown. (DOC) [file pone.0030410.s003.doc]

*Table S1. List of mitochondrial protein-coding genes mutS homolog msh1 sequences from previous studies used in phylogenetic analyses in the present study. Species, GenBank accession numbers, geographic origin, latitude and longitude, and collection date are also shown.*

| Species | Accession no. | Site | Latitude | Longitude | Date | GenBank # |
| --- | --- | --- | --- | --- | --- | --- |
| *Sinularia* sp. | NTM-C011203 | Jepara, Central Java Sea | 06°35.00'S | 110°39.00'E | 1992 | DQ280594 |
| *Dampia pocilloporaeformis* | NTM-C005805 | Rowley Shoals, WA, Australia | 17°07.70'S | 119°20.20'E | 1987 | DQ280593 |
| *Lobophytum* sp. | UF2883 | Ginowan City, Okinawa, Japan | 26°17'N | 127°43'E | 2004 | DQ280591 |
| *Lobophytum* sp. | UF2860 | Ginowan City, Okinawa, Japan | 26°17'N | 127°43'E | 2004 | DQ280590 |
| *L. pauciflorum* | UF2856 | Ginowan City, Okinawa, Japan | 26°17'N | 127°43'E | 2004 | DQ280577 |
| *L. crassum* | UF2933 | Ginowan City, Okinawa, Japan | 26°17'N | 127°43'E | 2004 | DQ280566 |
| *L. crassum* | NTM-C014514 | Gulf of Carpentaria, Australia | 12°05.66'S | 136°47.75'E | 2003 | DQ280562 |
| *L. compactum* | UF2848 | Ginowan City, Okinawa, Japan | 26°17'N | 127°43'E | 2004 | DQ280560 |
| *S. trocheliophorum* | UF2638 | Ginowan City, Okinawa, Japan | 26°17'N | 127°43'E | 2004 | DQ280550 |
| *S. crassocaule* | NTM-C013916 | Milne Bay, Papua New Guinea | 10°36.72'S | 152°32.29'E | 1998 | DQ280507 |
| *S. glaucum* A | NTM-C014002 | Konanda Reef, Vanuatu | 17°45.17'S | 168°17.28'E | 2000 | DQ280531 |
| *S. glaucum*  A | RMNH Coel33074 | Berau I., NE Kalimantan, Indonesia | 02°04.45'N | 118°24.11'E | 2003 | DQ280538 |
| *S. glaucum*  B | NTM-C013530 | Semporna I., Sabah, Malaysia | 04°38.06'N | 118°42.58'E | 1999 | DQ280528 |
| *S. glaucum*  C | NTM-C013974 | Mauritiu 11, Mauritius | 19°56.75'S | 57°37.24'E | 1999 | DQ280530 |
| *S. glaucum*  D | NTM-C014153 | Woodlark I., Papua New Guinea | 09°13.89'S | 152°25.56'E | 2002 | DQ280534 |
| *S. cinereum* | UF2849 | Ie I., Okinawa, Japan | 26°44.35'N | 127°48.61'E | 2004 | DQ280506 |
| *S. glaucum*  F | NTM-C013554 | Semporna I., Sabah, Malaysia | 04°34.12'N | 118°44.29'E | 1999 | DQ280529 |
| *S. elegans* | UF2637 | Sesoko I., Okinawa, Japan | 26°38'N | 127°52'E | 2004 | DQ280520 |
| *S. ehrenbergi* | NTM-C011208 | Jepara, Central Java Sea | 06°35.00'S | 110°39.00'E | 1992 | DQ280512 |
| *S. nanwanensis* | RMNH Coel33078 | Berau I., NE Kalimantan, Indonesia | 02°04.45'N | 118°24.11'E | 2003 | DQ280542 |
