# Supplementary material for: Soft Coral Sarcophyton (Cnidaria: Anthozoa: Octocorallia) Species Diversity and Chemotypes
Source: PLoS One. 2012 Jan 17;7(1):e30410. doi: 10.1371/journal.pone.0030410 (PMC3260304; doi:10.1371/journal.pone.0030410)
Supplement: Table S2 — List of internal transcribed spacer of ribosomal DNA (ITS-rDNA) sequences from previous studies used in phylogenetic analyses in the present study. Species, GenBank accession numbers, geographic origin, and host species are shown. (DOC) [file pone.0030410.s004.doc]

Table S2. List of internal transcribed spacer of ribosomal DNA (ITS-rDNA) sequences from previous studies used in phylogenetic analyses in the present study. Species, GenBank accession numbers, geographic origin, and host species are shown.

| Species | Accession no. | Site | Host |
| --- | --- | --- | --- |
| Symbiodinium sp. CcFIZ | AF195144 | West Pacific, Palau | *Corculum cardissa* |
| Symbiodinium sp. Sakura 3a | AB207185 | Sakurajima, Japan | *Zoanthus sansibaricus* |
| Symbiodinium sp. Kokubu 2d | AB190271 | Kokubu, Japan | *Zoanthus sansibaricus* |
| Symbiodinium sp. Kokubu 2c | AB190270 | Kokubu, Japan | *Zoanthus sansibaricus* |
| Amakusa I isolate 9 | AY186567 | West Pacific, Kyushu | *Plesiastrea versipora* |
| Symbiodinium_sp. 1591_type_C91 | AJ291519 |  |  |
| Symbiodinium_sp. TcFIZ | AF195157 | West Pacific, Palau | *Tridacna crocea* |
| Symbiodinium_sp. 1366_type_C92 | AJ291514 | Red Sea, Gulf of Elat | *Amphisorus hemprichii* |
| Symbiodinium_sp. Yakushima2b | AB190279 | Yakushima, Japan | *Zoanthus sansibaricus* |
| Symbiodinium_sp. 1675a | AJ311944 | West Pacific, Guam | *Porites rus.* |
