# Supplementary material for: Soft Coral Sarcophyton (Cnidaria: Anthozoa: Octocorallia) Species Diversity and Chemotypes
Source: PLoS One. 2012 Jan 17;7(1):e30410. doi: 10.1371/journal.pone.0030410 (PMC3260304; doi:10.1371/journal.pone.0030410)
Supplement: Table S3 — Collection information for specimens included in molecular phylogenetic clade. (DOC) [file pone.0030410.s005.doc]

Table S3. Collection information for specimens included in molecular phylogenetic clade.

|  | Accession no. | |  |  |
| --- | --- | --- | --- | --- |
| Specimen | *msh 1* | ITS - rDNA | Chemotype(s) | Species / Clade |
| Sunabe 1 | AB665461 |  | chemotype 1: 2*S*,7*S*,8*S*-sarcophytoxide | *Sarcophyton* sp / mix clade |
| Sunabe 1 col-1 |  | AB665603 | chemotype 2: 2*S*,7*R*,8*R*-sarcophytoxide |  |
| Sunabe 1 col-2 |  | AB665604 | chemotype 4: 7,8-epoxy-1,3,11-cembratrien-15-ol |  |
| Sunabe 1 col-3 |  | AB665605 |  |  |
| Sunabe 1 col-4 |  | AB665606 |  |  |
| Sunabe 1 col-5 |  | AB665607 |  |  |
| Sunabe 2 * | AB665466 |  | chemotype 2: 2*S*,7*R*,8*R*-sarcophytoxide | *Sarcophyton glaucum* / clade D |
| Sunabe 2 col-1 |  | AB665608 |  |  |
| Sunabe 2 col-2 |  | AB665609 |  |  |
| Sunabe 2 col-5 |  | AB665610 |  |  |
| Sunabe 2 col-7 |  | AB665611 |  |  |
| Sunabe 4 | AB665459 |  | no marker | *Sarcophyton elegans* |
| Sunabe 4 col-1 |  | AB665612 |  |  |
| Sunabe 4 col-2 |  | AB665613 |  |  |
| Sunabe 4 col-4 |  | AB665614 |  |  |
| Sunabe 4 col-5 |  | AB665615 |  |  |
| Sunabe 4 col-6 |  | AB665616 |  |  |
| Sunabe 5 * | AB665467 |  | chemotype 1: 2*S*,7*S*,8*S*-sarcophytoxide | *Sarcophyton glaucum* / clade D |
| Sunabe 5 col-6 |  | AB665617 | chemotype 2: 2*S*,7*R*,8*R*-sarcophytoxide |  |
| Sunabe 5 col-7 |  | AB665618 |  |  |
| Sunabe 5 col-9 |  | AB665619 |  |  |
| Sunabe 5 col-10 |  | AB665620 |  |  |
| Sunabe 6 * | AB665472 |  | chemotype 3: 2*S*,7*R*,8*R*-isosarcophytoxide | *Sarcophyton glaucum* / clade B |
| Sunabe 6 col-1 |  | AB665621 |  |  |
| Sunabe 6 col-2 |  | AB665622 |  |  |
| Sunabe 6 col-3 |  | AB665623 |  |  |
| Sunabe 6 col-4 |  | AB665624 |  |  |
| Sunabe 6 col-5 |  | AB665625 |  |  |
| Sunabe 7 | AB665451 |  | chemotype 1: 2*S*,7*S*,8*S*-sarcophytoxide | *Sarcophyton trocheliophorum* |
| Sunabe 7 col-1 |  | AB665626 |  |  |
| Sunabe 7 col-2 |  | AB665627 |  |  |
| Sunabe 7 col-3 |  | AB665628 |  |  |
| Sunabe 7 col-5 |  | AB665629 |  |  |
| Sunabe 10 | AB665462 |  | chemotype 1: 2*S*,7*S*,8*S*-sarcophytoxide | *Sarcophyton* sp / mix clade |
| Sunabe 10 col-3 |  | AB665630 | chemotype 8: 7*S*,8*S*-epoxy-1,3,11-cembratriene |  |
| Sunabe 10 col-4 |  | AB665631 |  |  |
| Sunabe 10 col-5 |  | AB665632 |  |  |
| Sunabe 12 | AB665447 |  | chemotype 1: 2*S*,7*S*,8*S*-sarcophytoxide | *Sarcophyton trocheliophorum* |
| Sunabe 12 col-6 |  | AB665633 |  |  |
| Sunabe 12 col-7 |  | AB665634 |  |  |
| Sunabe 12 col-9 |  | AB665635 |  |  |
| Sunabe 13 * | AB665470 |  | chemotype 1: 2*S*,7*S*,8*S*-sarcophytoxide | *Sarcophyton glaucum* / clade B |
| Sunabe 13 col-2 |  | AB665636 |  |  |
| Sunabe 13 col-3 |  | AB665637 |  |  |
| Sunabe 13 col-4 |  | AB665638 |  |  |
| Sunabe 13 col-5 |  | AB665639 |  |  |
| Sunabe 14 | AB665449 |  | chemotype 1: 2*S*,7*S*,8*S*-sarcophytoxide | *Sarcophyton trocheliophorum* |
| Sunabe 14 col-1 |  | AB665640 |  |  |
| Sunabe 14 col-2 |  | AB665641 |  |  |
| Sunabe 14 col-3 |  | AB665642 |  |  |
| Sunabe 14 col-4 |  | AB665643 |  |  |
| Sunabe 14 col-5 |  | AB665644 |  |  |
| Sunabe 15 | AB665452 |  | chemotype 1: 2*S*,7*S*,8*S*-sarcophytoxide | *Sarcophyton trocheliophorum* |
| Sunabe 15 col-1 |  | AB665645 |  |  |
| Sunabe 15 col-3 |  | AB665646 |  |  |
| Sunabe 15 col-5 |  | AB665647 |  |  |
| Sunabe 15 col-6 |  | AB665648 |  |  |
| Sunabe 15 col-7 |  | AB665649 |  |  |
| Sunabe 16 | AB665450 |  | chemotype 1: 2*S*,7*S*,8*S*-sarcophytoxide | *Sarcophyton trocheliophorum* |
| Sunabe 16 col-2 |  | AB665650 | chemotype 2: 2*S*,7*R*,8*R*-sarcophytoxide |  |
| Sunabe 16 col-9 |  | AB665651 |  |  |
| Sunabe 16 col-10 |  | AB665652 |  |  |
| Sunabe 17 * | AB665464 |  | chemotype 2: 2*S*,7*R*,8*R*-sarcophytoxide | *Sarcophyton glaucum* / clade D |
| Sunabe 17 col-1 |  | AB665653 |  |  |
| Sunabe 17 col-2 |  | AB665654 |  |  |
| Sunabe 17 col-4 |  | AB665655 |  |  |
| Sunabe 17 col-5 |  | AB665656 |  |  |
| Sunabe 17 col-6 |  | AB665657 |  |  |
| Sunabe 18 * | AB665469 |  | chemotype 1: 2*S*,7*S*,8*S*-sarcophytoxide | *Sarcophyton glaucum* / clade B |
| Sunabe 18 col-2 |  | AB665658 |  |  |
| Sunabe 18 col-8 |  | AB665659 |  |  |
| Sunabe 18 col-10 |  | AB665660 |  |  |
| Sunabe 19 * | AB665465 |  | chemotype 3: 2*S*,7*R*,8*R*-isosarcophytoxide | *Sarcophyton glaucum* / clade D |
| Sunabe 19 col-1 |  | AB665661 |  |  |
| Sunabe 19 col-3 |  | AB665662 |  |  |
| Sunabe 19 col-5 |  | AB665663 |  |  |
| Sunabe 20 | AB665448 |  | chemotype 1: 2*S*,7*S*,8*S*-sarcophytoxide | *Sarcophyton trocheliophorum* |
| Sunabe 20 col-1 |  | AB665664 |  |  |
| Sunabe 20 col-3 |  | AB665665 |  |  |
| Sunabe 20 col-4 |  | AB665666 |  |  |
| Sunabe 20 col-5 |  | AB665667 |  |  |
| Sunabe 21 | AB665446 |  | chemotype 1: 2*S*,7*S*,8*S*-sarcophytoxide | *Sarcophyton trocheliophorum* |
| Sunabe 21 col-2 |  | AB665668 |  |  |
| Sunabe 21 col-3 |  | AB665669 |  |  |
| Sunabe 22 * | AB665471 |  | chemotype 1: 2*S*,7*S*,8*S*-sarcophytoxide | *Sarcophyton glaucum* / clade B |
| Sunabe 22 col-6 |  | AB665670 |  |  |
| Sunabe 22 col-7 |  | AB665671 |  |  |
| Sunabe 22 col-10 |  | AB665672 |  |  |
| Sunabe 23 | AB665453 |  | chemotype 1: 2*S*,7*S*,8*S*-sarcophytoxide | *Sarcophyton trocheliophorum* |
| Sunabe 23 col-3 |  | AB665673 |  |  |
| Sunabe 23 col-4 |  | AB665674 |  |  |
| Sunabe 23 col-7 |  | AB665675 |  |  |
| Sunabe 23 col-8 |  | AB665676 |  |  |
| Sunabe 23 col-9 |  | AB665677 |  |  |
| Zanpa 1 | AB665455 |  | chemotype 1: 2*S*,7*S*,8*S*-sarcophytoxide | *Sarcophyton trocheliophorum* |
| Zanpa 1 col-6 |  | AB665678 |  |  |
| Zanpa 1 col-7 |  | AB665679 |  |  |
| Zanpa 1 col-8 |  | AB665680 |  |  |
| Zanpa 1 col-10 |  | AB665681 |  |  |
| Zanpa 3 * | AB665474 |  | chemotype 7: 7-hydroxy-1,3,11-cembratrien-20,8-olide | *Sarcophyton glaucum* / clade F |
| Zanpa 3 col-2 |  | AB665682 |  |  |
| Zanpa 3 col-3 |  | AB665683 |  |  |
| Zanpa 3 col-4 |  | AB665684 |  |  |
| Zanpa 3 col-5 |  | AB665685 |  |  |
| Zanpa 4 * | AB665475 |  | chemotype7:7-hydroxy-1,3,11-cembratrien-20,8-olide | *Sarcophyton glaucum* / clade F |
| Zanpa 4 col-10 |  | AB665686 |  |  |
| Zanpa 5 | AB665456 |  | chemotype 1: 2*S*,7*S*,8*S*-sarcophytoxide | *Sarcophyton trocheliophorum* |
| Zanpa 5 col-2 |  | AB665687 |  |  |
| Zanpa 5 col-3 |  | AB665688 |  |  |
| Zanpa 5 col-4 |  | AB665689 |  |  |
| Zanpa 5 col-5 |  | AB665690 |  |  |
| Zanpa 6 | AB665473 |  | chemotype 6: Emblide | Sarcophyton sp |
| Zanpa 6 col-1 |  | AB665691 |  |  |
| Zanpa 6 col-3 |  | AB665692 |  |  |
| Zanpa 6 col-4 |  | AB665693 |  |  |
| Zanpa 6 col-5 |  | AB665694 |  |  |
| Zanpa 6 col-6 |  | AB665695 |  |  |
| Zanpa 9 | AB665457 |  | chemotype 1: 2*S*,7*S*,8*S*-sarcophytoxide | *Sarcophyton trocheliophorum* |
| Zanpa 9 col-1 |  | AB665696 |  |  |
| Zanpa 9 col-3 |  | AB665697 |  |  |
| Zanpa 9 col-4 |  | AB665698 |  |  |
| Zanpa 10 * | AB665476 |  | chemotype 6: Emblide | *Sarcophyton glaucum* / clade F |
| Zanpa 10 col-1 |  | AB665699 |  |  |
| Zanpa 10 col-2 |  | AB665700 |  |  |
| Zanpa 10 col-3 |  | AB665701 |  |  |
| Zanpa 10 col-4 |  | AB665702 |  |  |
| Zanpa 10 col-5 |  | AB665703 |  |  |
| Mizugama 4 | AB665460 | - | chemotype 3: 2*S*,7*R*,8*R*-isosarcophytoxide | *Sarcophyton* sp / mix clade |
|  |  |  | chemotype 5: Sarcophytol A |  |
| Mizugama 5 | AB665478 | - | no marker | *Sarcophyton glaucum* / clade F |
| Mizugama 6 * | AB665463 |  | chemotype 1: 2S,7S,8S-sarcophytoxide | *Sarcophyton glaucum* / clade D |
| Mizugama 6 col-4 |  | AB665704 |  |  |
| Mizugama 6 col-5 |  | AB665705 |  |  |
| Mizugama 6 col-8 |  | AB665706 |  |  |
| Mizugama 6 col-9 |  | AB665707 |  |  |
| Mizugama 7 | AB665468 |  | chemotype 2: 2*S*,7*R*,8*R*-sarcophytoxide | *Sarcophyton glaucum* / cladeC |
| Mizugama 7 col-6 |  | AB665708 |  |  |
| Mizugama 7 col-9 |  | AB665709 |  |  |
| Mizugama 8 | AB665454 |  | chemotype 1: 2S,7S,8S-sarcophytoxide | *Sarcophyton trocheliophorum* |
| Mizugama 8 col-7 |  | AB665710 |  |  |
| Mizugama 8 col-8 |  | AB665711 |  |  |
| Mizugama 9 * | AB665479 |  | no marke | *Sarcophyton glaucum* / clade F |
| Mizugama 9 col-3 |  | AB665712 |  |  |
| Mizugama 9 col-7 |  | AB665713 |  |  |
| Mizugama 9 col-8 |  | AB665714 |  |  |
| Mizugama 9 col-9 |  | AB665715 |  |  |
| Mizugama 10 * | AB665477 |  | no marker | *Sarcophyton glaucum* / clade F |
| Mizugama 10 col-1 |  | AB665716 |  |  |
| Mizugama 10 col-2 |  | AB665717 |  |  |
| Mizugama 10 col-3 |  | AB665718 |  |  |
| Mizugama 10 col-4 |  | AB665719 |  |  |
| Mizugama 11 | AB665458 |  | chemotype 1: 2S,7S,8S-sarcophytoxide | *Sarcophyton trocheliophorum* |
| Mizugama 11 col-1 |  | AB665720 |  |  |
| Mizugama 11 col-3 |  | AB665721 |  |  |
| Mizugama 11 col-4 |  | AB665722 |  |  |
| Mizugama 11 col-5 |  | AB665723 |  |  |

Note: Images of sclerites for all specimens were taken. * Specimens for which sclerites were examined. Specimens were named by place, colony, clone No.
